# Supplementary material for: Wall Teichoic Acids Facilitate the Release of Toxins from the Surface of Staphylococcus aureus
Source: Microbiol Spectr. 2022 Jul 7;10(4):e01011-22. doi: 10.1128/spectrum.01011-22 (PMC9430763; doi:10.1128/spectrum.01011-22)
Supplement: Supplemental file 1 — Supplemental material. Download spectrum.01011-22-s0001.pdf, PDF file, 1.6 MB [file spectrum.01011-22-s0001.pdf]

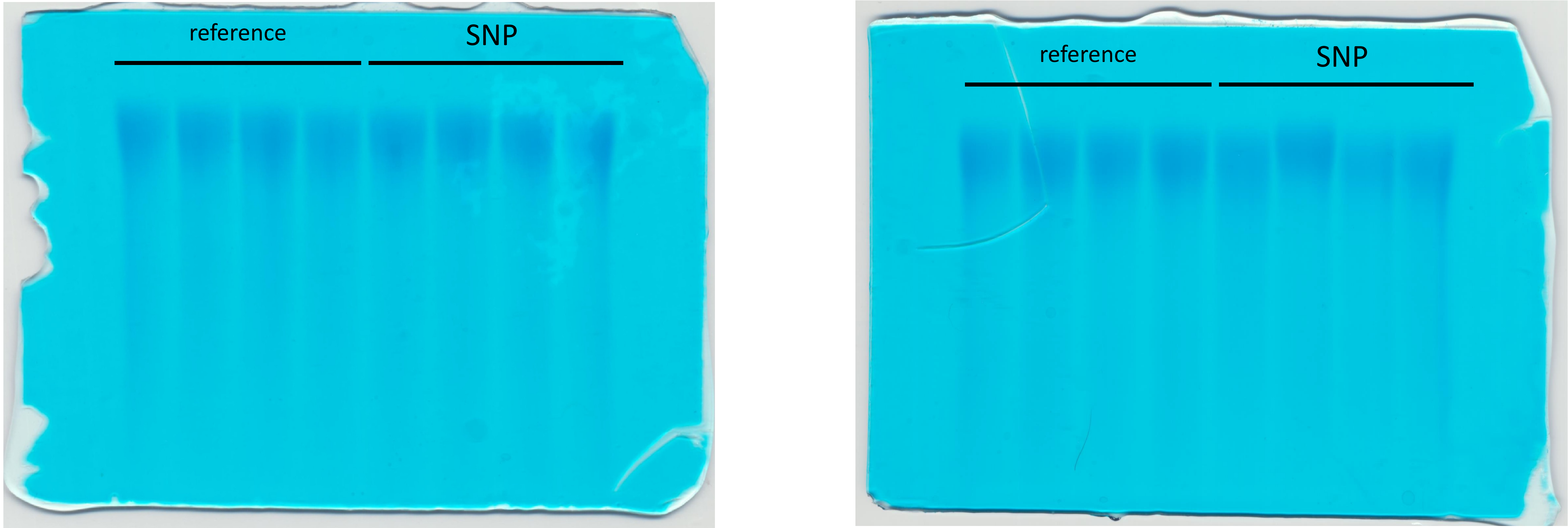

Supp. Fig. 1: The presence of the intergenic SNP does not affect WTA length/polymerisation. WTA was extracted from 16 clinical isolates, eight with the reference (wild type) *tar* intergenic sequence, and eight with the intergenic SNP. No difference in the length or polymerisation of the WTA was evident.

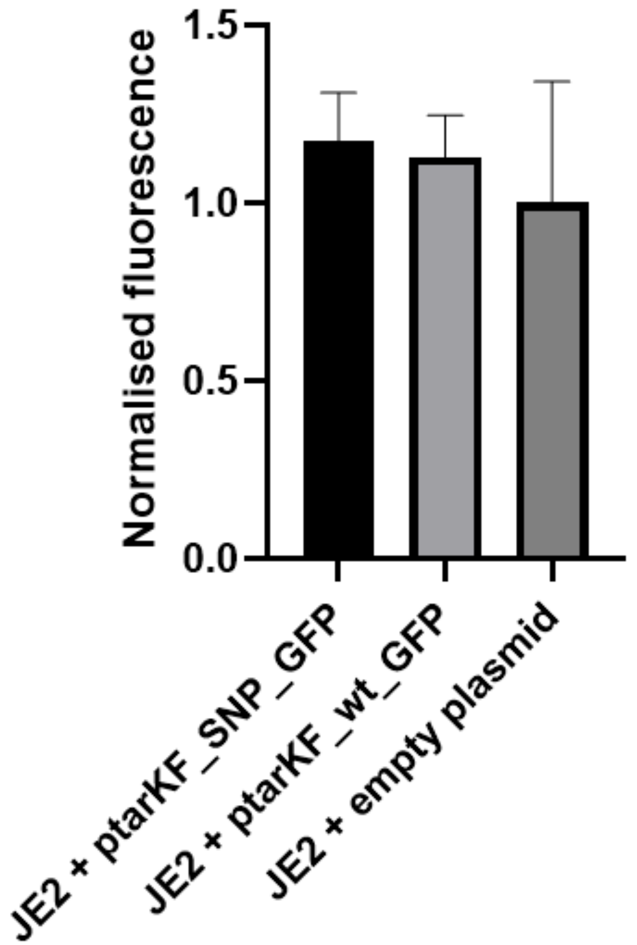

Supp. Fig. 2: Transcription from the *tarF* promoter is undetectable by promoter GFP fusion. The graph shows the fluorescence detected from overnight growths of JE2 transformed with pOS1 plasmid containing the GFP reporter fusion with the wild type (wt) intergenic region, pOS1 plasmid containing the GFP reporter fusion with the SNP intergenic region and empty pOS1 plasmid. Fluorescence values were normalised to the empty plasmid control.

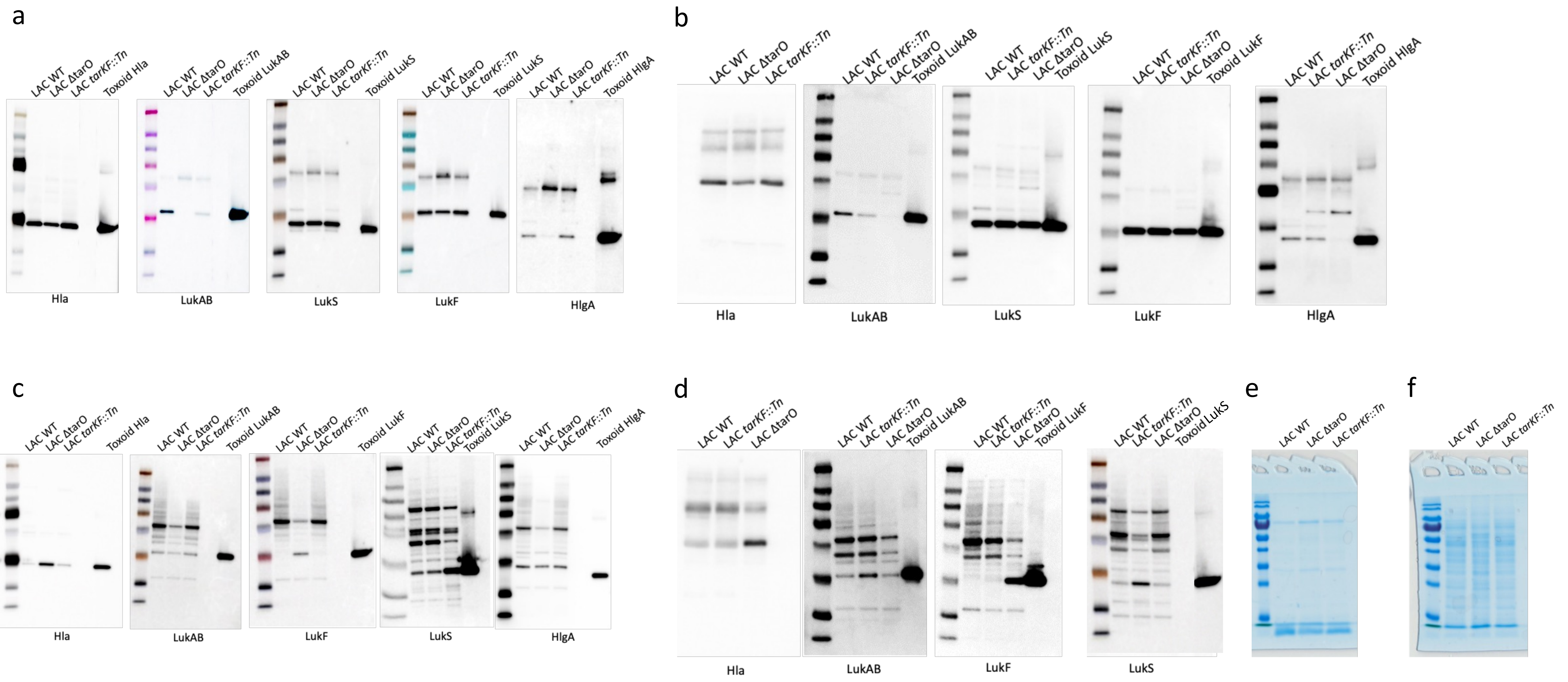

Supp Fig. 3: Toxins abundance in whole cell lysates and supernatants replicates. Western replicates (a) supernatant, replicate 1. (b) supernatant, replicate 2. (c) whole cell lysate, replicate 1. (d) whole cell lysate, replicate 2. Since HlgA was not detectable in the first replicate, the western was not repeated. Coomassie gels replicates (e) supernatants, replicate 2. (f) whole cell lysate, replicate 2.

Accession	Description	Score	Coverage	Area	# AAs	MW [kDa]
Q2FWM8	Delta-hemolysin	26.65	96.15	3.782×10^{7}	26	3.0
Q2FZA4	Phenol soluble modulin beta 1	13.75	70.45	8.575×10^{6}	44	4.5
P0C7Y1	Phenol-soluble modulin alpha 1 peptide	4.68	85.71	9.601×10^{7}	21	2.3
P0C818	Phenol-soluble modulin alpha 4 peptide	4.32	85.00	1.680×10^{8}	20	2.2
Q2FZA3	Phenol soluble modulin beta 2	4.17	40.91	4.538×10^{5}	44	4.5

Supp. Table 1. PSMs band identification. The table shows the proteins identified in the 3kDa band on SDS page gel from JE2 supernatants. Coverage: percentage of the protein sequences covered by identified peptides. Areas: distribution of areas calculated by the Peptide and Protein Quantifier node over the sample groups. # Aas: the theoretical length of the protein sequence. MW [kDa]: theoretical molecular weight of the protein.

Toxin	isoelectric point
LukG	8.42
LukH	9.30
Hla	7.94
LukS	8.90
LukF	8.96
HlgA	9.53
Hld	9.03
Psmα1	9.70
Psmα2	10.00
Psmα3	9.52
Psmα4	9.70
Psmβ1	4.78
Psmβ2	5.36

Supp. Table 2. Isoelectric points of the toxins whose secretion is impaired in a WTA mutant. The isoelectric points were calculated on the amino acid sequence, excluding the signal peptide, using the online tool Compute pI/Mw (https://web.expasy.org/compute_pi/)**.**

Supp. Table 2. Clinical strains used in this study. The strain names for 90 isolates of *S. aureus* of the ST239 lineage are listed. Whether they contain the *tarF* promoter SNP is indicated, as is their toxicity as measured by vesicle lysis.

Strain ID	Tarf SNP?	Toxicity (fluor.units)
IU12	SNP	86313
IU9	SNP	85826
IU4	SNP	85146
IU17	SNP	85090
IU20	SNP	83862
HU13	SNP	83420
HU7	SNP	82231
HU8	SNP	82125
DEU29		81384
HU17	SNP	80774
MU11	SNP	79495
IU10	SNP	72244
DEU20		62940
DEU17		62740
DEU37		62085
DEU9		60435
HU16		59307
HU11		58291
HU5		52212
HU6		46616
HU4		40677
DEU42		34291
DEU3		33352
DEU40		33022
DEU1		31815
DEU27		28779
DEU30		27803
DEU4		27328
IU1		27103
DEU7		25698

Strain ID	Tarf SNP?	Toxicity (fluor. units)
IU19		25421
DEU6		25366
DEU25		25121
DEU8		23850
IU6		22072
DEU15		20765
DEU23		20679
MU1		20127
DEU41		19862
MU19		19774
IU16		19587
IU7		19443
HU9		18330
DEU19		17354
DEU11		17264
DEU39		17119
MU20		16907
DEU16		16780
DEU28		16752
IU13		16622
IU3	SNP	16590
DEU38		16316
DEU14		16179
HU12		16062
IU18		16053
DEU50		16017
MU3		15943
IU14		15893
IU2	SNP	15832
IU15		15793

Strain ID	Tarf SNP?	Toxicity (fluor.units)
DEU12		15424
DEU49		15328
HU19		15119
DEU5		14901
IU11	SNP	14864
DEU22		14845
HU21		14826
DEU35		14607
DEU36		14555
DEU46		14341
HU14		13637
MU2		13452
DEU47		13390
MU6		13264
IU8		12317
DEU10		11988
DEU2		11827
HU18		11402
HU2		11383
HU15		11299
MU4	SNP	10580
DEU43		10115
MU10		9426
HU10		9426
MU7		8989
MU5		8829
HU23		7859
MU9		7567
HU26		5049
HU24		4693
